# A mentally healthy framework to guide employers and policy makers

**DOI:** 10.3389/fpubh.2024.1430540

**Published:** 2024-07-22

**Authors:** Mark Deady, Samineh Sanatkar, Leona Tan, Nick Glozier, Aimee Gayed, Katherine Petrie, Vita Ligaya Dalgaard, Elizabeth Stratton, Anthony D. LaMontagne, Samuel B. Harvey

**Affiliations:** ^1^Faculty of Medicine and Health, Black Dog Institute, UNSW Sydney, Randwick, NSW, Australia; ^2^Faculty of Medicine and Health, Central Clinical School, University of Sydney, Camperdown, NSW, Australia; ^3^Australian Research Council (ARC) Centre of Excellence for Children and Families Over the Life Course, Sydney, NSW, Australia; ^4^Faculty of Medicine and Health, University of New South Wales, Sydney, NSW, Australia; ^5^Department of Psychology and Behavioral Sciences, School of Business and Social Sciences, Aarhus University, Aarhus, Denmark; ^6^Institute for Health Transformation, School of Health and Social Development, Deakin University, Geelong, VIC, Australia

**Keywords:** work stress, occupational mental health, return to work, psychological hazards, employee wellbeing, intervention, mental health, workplace

## Abstract

Mental health problems among the working population represent a growing concern with huge impacts on individuals, organizations, compensation authorities, and social welfare systems. The workplace presents both psychosocial risks and unique opportunities for intervention. Although there has been rapid expansion of workplace mental health interventions over recent decades, clear direction around appropriate, evidence-based action remains limited. While numerous workplace mental health models have been proposed to guide intervention, general models often fail to adequately consider both the evidence base and where best-practice principles alone inform action. Further, recommendations need to be updated as new discoveries occur. We seek to update the *Framework for Mentally Healthy Workplaces* based on new evidence of intervention effectiveness while also incorporating evidence-based principles. The updated model also integrates concepts from existing alternate models to present a comprehensive overview of strategies designed to enhance wellbeing, minimize harm, and facilitate recovery. Examples of available evidence and obstacles to implementation are discussed. The Framework is designed to support employers and managers in determining which strategies to apply and to guide future avenues of research.

## Background

Employee mental health and wellbeing is arguably the most significant public health issue facing modern workplaces (1), affecting both high- and low-income countries (2). Leaders needing to think about employee mental health is no longer extraneous to business considerations or only the realm of select industries or organizations. Apart from the personal consequences of psychological injury, the costs associated with productivity loss, turnover, absenteeism, and healthcare (e.g., compensation, early retirement payouts) make this issue not only a social, but a financial imperative for businesses (3). Workplace safety is often defined around hazards (aspects that have the potential to cause harm) and risk (encompasses the probability of exposure and the extent of damage). Increasing focus on these areas in terms of mental health is occurring with major focus on psychosocial risk in the workplace.

## Psychosocial risk at work

**Figure 1:**
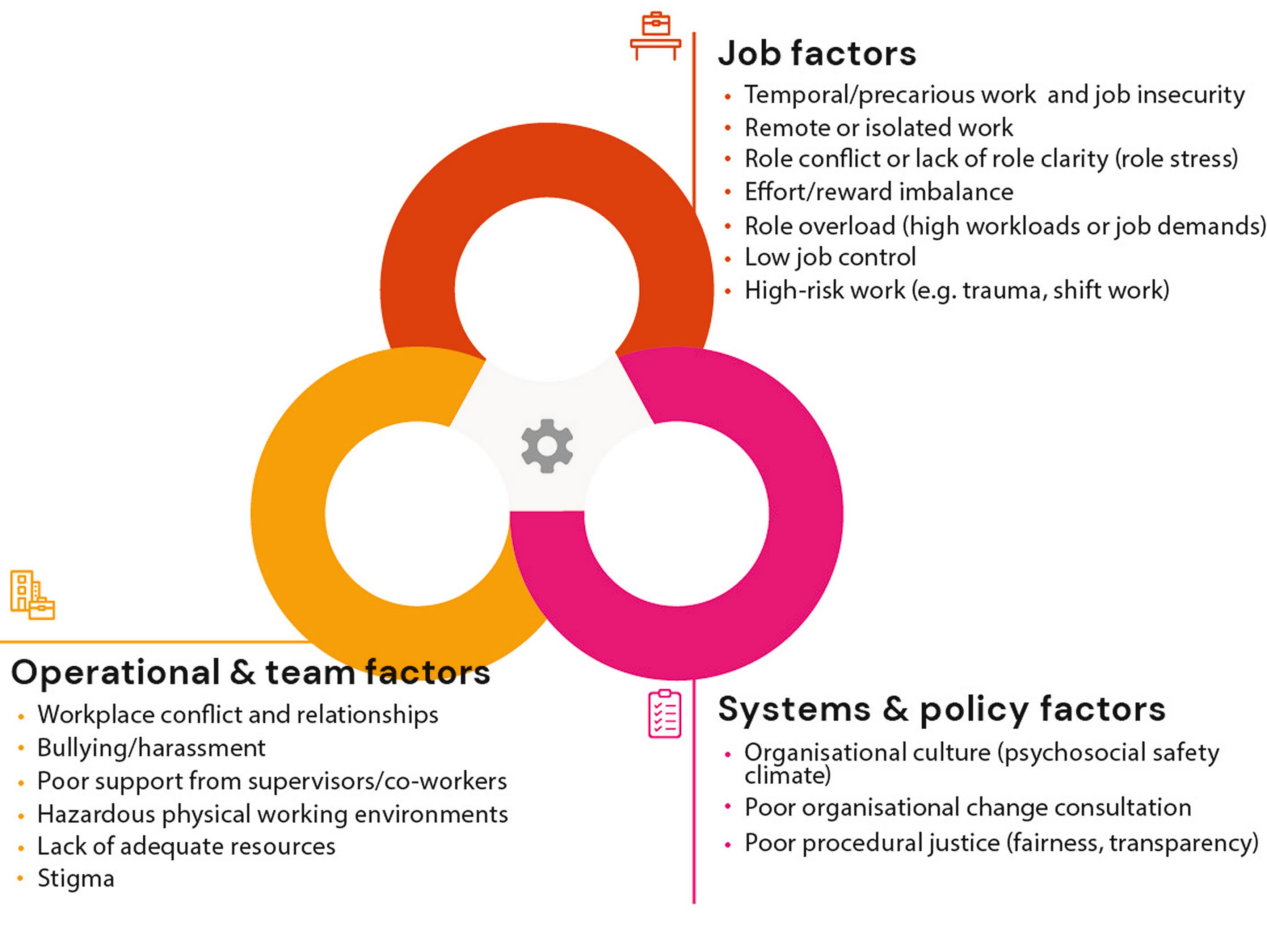
Breakdown of established psychosocial risk factors.

There is strong evidence linking specific workplace factors with poor mental health outcomes. These factors relate to: (i) the design and content of the work being conducted (job factors), (ii) the environment in which this occurs (operational and team factors), and/or (iii) the wider context and culture of an organization (systems and policy factors; Figure 1). Specifically these include job demands (e.g., monotony, workload, hours) (4–7), role factors (e.g., role clarity, role conflict) (8), job control (e.g., flexibility, autonomy) (4, 5, 9), routine exposure to high-risk situations (e.g., trauma, shift work) (10–13), recognition and job stability (e.g., insecurity, effort-reward imbalance) (14, 15), interpersonal relationships (e.g., lack of support, conflict, bullying and harassment) (16, 17), and organizational justice and culture (5, 16, 18, 19). In addition to an increased understanding of workplace risk factors, research efforts have produced a growing evidence-base for workplace interventions that can either modify or mitigate these risk factors (20).

## Previous workplace mental health frameworks, models, and guidelines

Greater understanding of the role workplaces can play in improving population mental health has led to specific evidence-based framing and guidance in the management of risk and response to distress, illness, and incapacity. Some noteworthy examples include the *Integrated Intervention Approach* (21), the *World Health Organisation’s (WHO) Guidelines on Mental Health at Work* (2), and *Thrive at Work Integrative Framework* (22, 23).

Our own model, the *Framework to Create Mentally Healthy Workplaces* (18, 24) was based on a significant review of the available evidence base (20) and outlined the role of workplace interventions in addressing worker mental health according to five broad areas: (1) Designing work to minimize harm, (2) Building organizational resilience through good management, (3) Enhancing personal resilience, (4) Promoting and facilitating early help-seeking, and (5) Supporting recovery and return to work. Three major developments have since occurred to suggest that an update to this framework is required. Firstly, in 2022, the WHO produced their first ever evaluation and guidance for mental health at work (2). This, together with our own updated meta-review of the evidence base (25), highlights important new knowledge [e.g., (26)] that should be translated into guidance for employers and policy makers. Secondly, national and international regulatory and legislative changes (27) have led to shifts in employer responsibilities regarding mental health at work. Thirdly, there is an opportunity to bring together some of the concepts and perspectives captured in the various workplace mental health frameworks into one unifying model.

This paper aims to provide an update of this prior framework that can be applied by organizations, guide managers and policy makers, and highlight important areas for further research. The updated Framework presents a synthesis of the major workplace models, illustrating which strategies and interventions are indicated for employees with different mental health and wellbeing support needs and the different levels of an organization where interventions can be conducted.

## Updated framework to create mentally healthy workplaces

**Figure 2:**
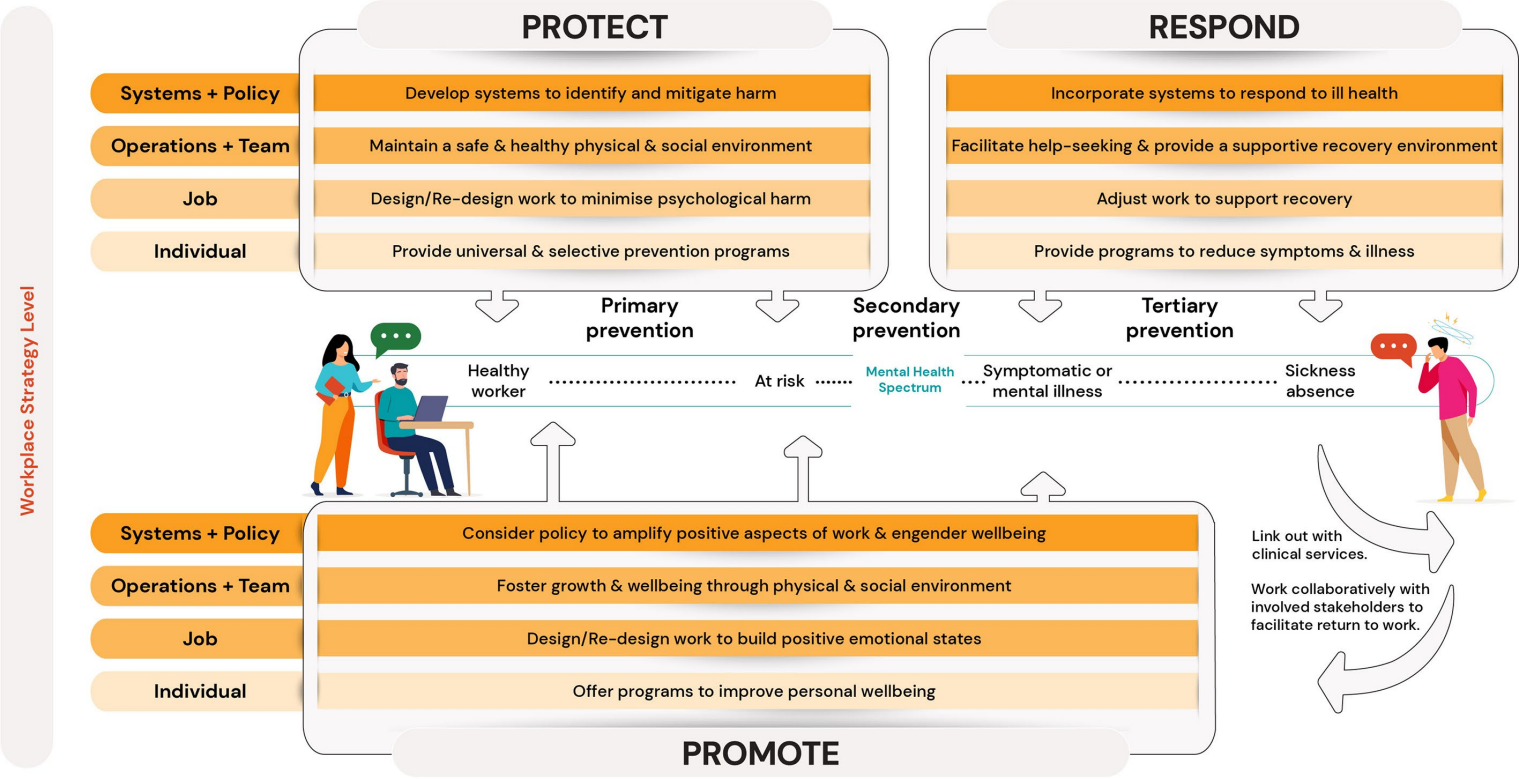
Updated Framework to Create Mentally Healthy Workplaces.

We considered two sources of evidence for the Framework update (Figure 2). Firstly, we sighted primary, secondary, and tertiary level *evidence from specific programs or interventions* which are rolled out to reduce risk, mental ill health, or to improve wellbeing. Secondly, we sighted *evidence-informed principles* underpinning mental health and wellbeing strategies within organizations. This more complete approach expands on the original Framework (24) in order to enhance recommendations for action where the most rigorous of evaluations are complex, and thus, lacking.

The updated Framework looks to highlight where strategies aim to deliver benefit on an individual worker’s mental health spectrum (i.e., during periods of healthy working life, in the early stages of injury or illness, or once an injury or illness is present). To this end, we adopted the Protect, Respond, and Promote pillar classifications (21, 28). Protect strategies are those primary or universal prevention interventions or initiatives in order to mitigate hazards and minimize harm or psychosocial injury. Promote strategies go beyond harm minimization and hazard mitigation and toward enhanced wellbeing and thriving. Respond strategies are those interventions that are delivered in response to a worker experiencing distress, and tertiary interventions aimed at workers who are unwell (either at-work or on leave) or returning from sick-leave. Where Protect and Respond operate at distinct ends of the mental health spectrum, promote strategies are likely to hold benefit to all workers regardless of where on the spectrum they lie, however, primary emphasis is on the early stages in the mental health spectrum.

Four workplace strategy levels provide guidance around where, within an organization, engagement is directed. These workplace strategy levels were designed to align with categories of workplace risk factors (Figure 1). The *Systems and Policy level* comprises of the policies or procedural arrangements operating within an organization. The *Operations and Team level* encompasses initiatives aimed at optimizing the interpersonal, team, and general environment in which work occurs. The *Job level* refers to initiatives to alter the design, delivery, or content of the work tasks being performed. The *Individual level* encapsulates those programs delivered to employees in order to modify perceptions and responses to conditions/experiences rather than via workplace changes. This 4-level conceptualization expands on the previous structure of organization, team, and individual levels.

In contrast to the original framework, the major changes are as follows: (i) incorporation of promotional pillar; (ii) finer delineation of the four strategy levels of intervention; (iii) richer consideration of *evidence-informed principles* underpinning strategies within organizations; (iv) update based on new evidence. The updated Framework was developed and is described in a way that intends to allow for variation depending on industry or size, while providing a unifying model and an evidence-based to integrate workplace mental health considerations, strategies, and interventions. The following sections synthesize the available evidence under each of these three pillars (Protect, Respond, Promote) across the four levels (Systems and Policy, Operations and Team, Job, and Individual).

## Summary of the evidence base

While this framework attempts to summarize the available evidence (25), it is important to highlight a disconnect that can occur between mentally healthy workplaces as defined in academic literature, and the reality of what employers are able to achieve (18). We have attempted to accommodate this research evidence gap by including some recommendations based on evidence-informed principles even if intervention trial data is lacking. In some cases, rigorous evaluation via traditional means is difficult, this is particularly true for many systems and operational interventions, we discuss this in turn. Table 1 summarizes some notable examples of workplace mental health strategies and the current level of evidence available for each of them.

**Table 1:** Summary of evidence and principles to support and enhance workplace mental health.

	Protect against harm	Promote good health	Respond to ill health
Systems and policy	*Develop systems to identify and mitigate harm*	*Consider policy to amplify positive aspects of work and engender wellbeing*	*Incorporate systems to respond to ill health*
	Psychosocial risk assessment and management ^c^ Change management procedures ^c^ Provisions to uphold organizational justice ^c^	Comprehensive cross-systems models that influence job design and introduce a range of other employment welfare practices ^a^	Equity, diversity, and inclusion policies ^c^ Return to Work programs ^b^ Facilitation of access to quality clinical care ^c^
Operational and team	*Maintain a safe and healthy physical and social environment*	*Foster growth and wellbeing through physical and social environment*	*Facilitate help-seeking and provide a supportive recovery environment*
	Preventative manager training ^b^ Mitigate hazardous physical conditions ^c^ Antibullying programs ^c^	Facilitated socialization (e.g., team cohesion programs, physical spaces) ^c^ Consideration of wellbeing in design of physical spaces ^b^	Responsive manager training ^a^ Supervisor support during recovery ^b^ Peer support programs ^b^ MH awareness/anti-stigma initiatives ^b^
Job	*Design/Re-design work to minimize psychological harm*	*Design/Re-design work to build positive emotional states*	*Adjust work to support recovery*
	Improve job control ^b^ Mitigate high-risk work ^b^ Increase job security ^c^ Limit long hours ^c^ Address effort-reward imbalance ^c^	Enhancements to worker autonomy ^b^ Job crafting ^b^ Comprehensive interventions addressing material, organizational, and working time-related conditions simultaneously ^a^ Employee participatory interventions ^b^	Appropriate work adjustments ^b^ Adjustments to work schedule and provision of additional support ^a^
Individual	*Provide universal and selective prevention programs*	*Offer programs to improve personal wellbeing*	*Provide programs to reduce symptoms and illness*
	Mindfulness programs ^a^ CBT programs ^a^ Physical health programs ^b^ Pre/post trauma exposure programs ^b^ Psychological first aid ^b^	Mindfulness programs ^a^ Positive psychology and resilience programs ^a^ CBT programs ^a^ Physical health programs ^b^ Individual coaching programs ^c^	Facilitated access to evidence-based care ^a^ CBT programs (face-to-face or digitally) ^a^ Employee Assistance Programs ^b^ Indicated physical exercise and/or mindfulness programs ^b^

CBT, Cognitive behavior therapy; MH, Mental health. ^a^Consistent evidence supporting effect of interventions. ^b^Limited evidence for specific interventions but sound principles for recommendation. ^c^Recommendation made solely on principles with little or no research evidence for specific interventions.

## Protect

“Protect” strategies involve the identification and management of work-related hazards to reduce risks to mental health to prevent and minimize harm or injury. Employers are obligated to take reasonable steps to identify and manage psychosocial hazards (along with physical, chemical, biological, radiological, ergonomic ones). Thus, compliance with legal obligations related to work health and safety, workers’ compensation, workplace relations, privacy and discrimination laws are fundamental. Relevant strategies and interventions are recommended across workplaces to meet these obligations.

### Systems and policy level: develop systems to identify and mitigate harm

This category includes the systems and policies built into an organization which reflect the investment in protecting worker mental health. Where these policies aim to optimize the salutogenic aspects of the job, they are discussed in the Promote pillar, while risk management would fall under Protect and Respond. This level subsumes the broader regulatory framework within which most organizations operate. In many jurisdictions, legislative amendments and codes of practice oblige employers to implement psychosocial work factors into occupational health and safety risk assessments [e.g., (29)]. This is exemplified by the International Labor Organization’s guidance for businesses (30) suggesting prevention of psychosocial workplace risk requires:

- Implementation of collective risk assessment and management/control measures;
- Increasing the coping ability of workers via job control increases;
- Improving organizational communication;
- Allowing workers’ participation in decision-making;
- Building social support systems within the workplace;
- Consideration of working and living conditions;
- Optimize safety and health culture within the organization.

A typical risk assessment captures what could cause harm (hazard analysis), the impact of this harm along with current precautions implemented (risk evaluation), and the implementation, monitoring and reviewing of elimination/mitigation strategies (31). This method, however, has been criticized as inadequate for psychosocial risks (32), particularly due to difficulty determining critical exposure levels. Due to this complexity, several tools for psychosocial risk assessment have been developed in recent years. A recent review identified 10 different tools of this kind (33). A lack of usage of observational methods (e.g., worker surveys) and a lack of guidance around corrective actions for employers constituted the most significant limitations of the reviewed tools.

In addition to the way organizations respond to the regulatory requirements, systems/policies should also reflect the cultural beliefs and values held regarding employee wellbeing and the prioritization of psychological health within that organization. In many cases these systems/policies provide the means for implementation of practices at various other levels (e.g., flexible working policies, procedural fairness standards). In other cases, these systems/policies are processes to better engage or communicate with workers (e.g., improving change management procedures, avenues for worker feedback/participation).

### Operational and team level: maintain a safe and healthy physical and social environment

The operational and team level incorporates initiatives to prevent illness, injury, and minimize hazards and associated risks of psychological harm through improving the interpersonal or physical environment in which work occurs. To mitigate interpersonal harms of bullying and harassment, anti-bullying interventions have been developed. Unfortunately, there is currently little evidence of the effectiveness of any program of this nature despite the significant negative impact of this psychosocial hazard (34).

It is well-established that managers have considerable influence on employee mental health (35), and as such the potential benefits of effective manager mental health training has attracted increased attention. Evidence suggests that skill-based training for managers can improve knowledge, attitudes, and self-reported behavior in supporting employees experiencing mental health problems (36). Evaluation of preventative programs and employee-level outcomes are limited, however, preliminary findings are encouraging.

Physically unsafe environmental factors (e.g., faulty machinery, chemical exposure, presence of spills) have direct and indirect consequences for employee mental health (37). Recommendations focus on risk management and mitigation of such hazards, however, research in this space is limited. Relevant physical work environment factors include ambient elements (e.g., noise, temperature, air quality), spatial arrangements (e.g., layout, level of enclosure), architectural design (e.g., lighting, natural light, lack of privacy and comfort) and equipment (e.g., ergonomics, safety equipment) (38, 39). Interventions to modify the physical environment include furniture or structural barriers, break-out room space, noise canceling headphones, and lighting systems that alert staff to high noise volume in shared spaces (40). Overall, rigorous evaluation of these interventions in improving mental health outcomes is lacking.

### Job level: design and re-design jobs to minimize psychological harm

Job design is an ongoing process of review and redesign where jobs are continually reshaped in response to internal and external considerations. One of the most widely researched aspects of job design is job strain, which describes high work demands combined with low decision latitude. There is strong evidence that workers who report job strain will experience poorer mental health over time compared to those who do not (41). A range of strategies have been developed to enhance employees’ decision latitude, such as problem-solving committees, health promotion and training workshops, facilitated employee control over job tasks, and stress management committees (42). While several studies demonstrate that improving worker autonomy can be protective and result in reduced psychological distress, the evidence for any specific type of participatory intervention is weak (42). Increased employee discretion may lead to negative consequences in some circumstances, for example, when increased autonomy leads to task overload and role ambiguity (43). There is evidence to suggest certain flexible working arrangements (including, worktime control, working from home) may increase employees’ control, but the direct mental health consequences are less clear (44). There is some preliminary evidence around the use of capacity-building workshops and action plans to reduce work and organizational stressors to improve morale and absenteeism, but broader effects on organization-wide improvements are lacking (45).

Emerging evidence holds promise for multidimensional job design interventions to help improve physical and psychological demands, emotional exhaustion, and social support (46, 47). The SMART work design model aims to prevent harm by increasing job resources and reducing adverse job demands (48). The model first looks to assess—both from the perspective of workers and the organizational practices—an organization’s job design and provide guidance for restructuring of roles, activities, and responsibilities. Recommendations for workplace adjustments consider the need and the readiness of the organization to make certain changes (23). Trial level evaluation of the model is limited but there is considerable evidence around the principles upon which the model is based (49) and there is substantial application of the model in practice.

### Individual level: provide universal and selective prevention programs

Within the Protect pillar, individual-level interventions incorporate initiatives delivered to employees to prevent illness at a universal or selective level. There is considerable overlap with programs to enhance wellbeing and improve resilience (Promote pillar). Equally, many of these programs utilize theoretical underpinnings that also underlie indicated prevention and treatment programs (Respond pillar).

#### Universal prevention programs

Primary prevention programs are those offered to all individuals (regardless of health status) to better withstand the adverse impacts of stressors, preventing mental illness symptom development. In the workplace context, the most widely evaluated interventions are based on cognitive behavior therapy (CBT), stress management, or mindfulness principles. There is evidence to show that universal CBT-based programs prevent depressive symptoms in workers, albeit with small effect sizes (50, 51). In many cases, these programs are delivered digitally and have been associated with mild to moderate improvements of experiences of stress, depression, and anxiety (52–54). A metareview of a range of preventative workplace programs found good evidence for universal prevention interventions focused on CBT, mindfulness, or stress management strategies (55). These programs yielded moderate and large effects on mental health and quality of life outcomes. Mindfulness and contemplative interventions specifically yielded moderate effects on general distress. Mindfulness- and Acceptance and Commitment Therapy (ACT)-based programs have been found to be efficacious in reducing employee stress, psychological distress, burnout, poor sleep, and anxiety symptoms (56, 57), and when delivered digitally (53, 54). Programs between 1 and 4 sessions, or short-term engagement with longer programs, appear to have little effect (58).

Physical activity programs remain popular initiatives to protect against mental illness and improve mental health outcomes within organizations despite inconsistent evidence for their effectiveness, especially for workplace exercise programs (59). A related body of research has focused on the benefits of yoga or tai chi for stress or anxiety. These programs have most reliable effect where participants commit to at least 12 h of practice (60).

#### Selective prevention programs

Selective prevention interventions are aimed at employees at specific risk, with much work focused on high-stress, high-hazard, and high-risk occupations (e.g., first responders, military personnel, healthcare workers). Most programs have utilized CBT or mindfulness techniques (46, 61–63), with some evidence supporting physical activity programs to improve mental health outcomes (64). A recent umbrella review of 16 meta-analyses found that psychosocial selective prevention interventions were associated with small and moderate effects on depression, anxiety, and stress outcomes (55). Mindfulness-based interventions were associated with small effects for general distress and burnout, and with moderate and large effects on depression and stress and anxiety (55).

Many selective prevention interventions target the effects of work-related exposure to traumatic events (65). Post-incident interventions show the strongest support for the utility of trauma-focused CBT for individuals with a diagnosis of acute stress disorder (65). Team-based skills training has also been shown to reduce poor mental health symptoms after major incidents (66). Emerging evidence suggests the potential of mind–body exercise programs for trauma-exposed workers (67). Pre-incident interventions include pre-stress inoculation training (68) and attention bias modification training for the prevention of posttraumatic stress disorder (PTSD) (69, 70). Stress inoculation training is a form of CBT aimed at shifting negative bodily and mental reactions to stressors, helping the person cope with and manage difficult emotions (via direct [e.g., problem solving], and indirect [e.g., self-talk, breathing exercises] strategies). Attention bias modification training is a process to modify attention to attend to specific target stimuli and ignore others, attempting to correct attentional biases inherent in certain disorders. For pre-incident interventions, however, strong evidence is lacking. It should be noted that most trauma-focused selective prevention intervention studies engage military personnel, which limits the generalizability of findings to other occupational groups.

The importance of a strong evidence-base for initiatives to mitigate risk and protect trauma-exposed workers from harm has been underscored by the practice of psychological debriefing (following critical incident exposure). Importantly, research has consistently found such practices to be of minimal benefit and potential harm (71). Current guidelines discourage this form of debriefing, with a focus instead on watchful waiting (71) and practical support (72). Psychological first aid (PFA) has also become a popular intervention following exposure to conflict or disaster, emphasizing reduction of initial distress, addressing of basic needs, and promoting adaptive coping. Early studies have suggested PFA is a helpful and safe alternative to debriefing (73). As much as possible, workplaces should seek to increase social support and reduce work pressures in the period following exposure to traumatic events.

## Promote

While most emphasis is placed on harm prevention and response (due in part to regulatory considerations), there is growing interest in how positive aspects of work and strengths-based approaches can facilitate sustained and enhanced wellbeing outcomes (43). Traditionally, attention within workplace to mental health (where it exists) has been driven by an illness ideology (identifying risk factors for mental illness, underlying mechanisms, and appropriate intervention), with little attention given mental *health* and wellbeing and means to promote it (74). This is driven by the common conflation of the absence of mental illness with true mental health (74, 75), and further compounded by the regulatory and liability considerations of illness and injury. The Promote pillar captures initiatives that enhance the positive aspects of work as well as worker strengths and capacities in pursuit of positive outcomes (e.g., joy, engagement, job satisfaction). Wellbeing is a central concept in the Promote pillar with social, psychological, spiritual, and physical dimensions are all relevant (76). Traditionally, definitions of wellbeing tended to focus on psychological wellbeing as a composition of autonomy, positive relations with others, purpose in life, self-acceptance, environmental mastery and personal growth (77). In recent years, there has been a shift on PERMA theory of wellbeing encompassing aspects of both subjective wellbeing (positive emotion, meaning) and psychological wellbeing (engagement, relationships, accomplishment) (78). Thompson and Marks (79) suggest the wellbeing can be conceptualized as a dynamic process which asserts that individuals can flourish and experience subjective wellbeing through personal resources (e.g., optimism, self-esteem), appropriate external conditions (e.g., safe, secure, suitable environments), and psychosocial engagement (e.g., social connection, autonomy, respect). Organizations have capacity to influence these domains through policies, practices, design, and intervention. Workplace research in this space often centers on the concept of “thriving,” which is positively associated with affect, work engagement, and a range of social and health-related outcomes (80).

### Systems and policy level: consider policy to amplify positive aspects of work and engender wellbeing

To reflect the shift in mental health conceptualizations toward a more holistic regard for a person’s overall wellbeing (81), this category encapsulates policies and practices that strive to enhance positive wellbeing, optimize the benefits of work, or promote working within a strengths-based approach. Comprehensive programs that integrate both organizational and individual approaches such as Total Worker Health (TWH) have demonstrated positive impact on wellbeing outcomes (82, 83). The TWH approach incorporates programs in line with a hierarchy of controls around elimination/control of hazards, substitution of practices, work redesign, education, and individual change. Similarly, a review of 33 studies utilizing system-wide approaches that simultaneously enhance job design and introduce a range of other employment welfare practices concluded that such programs were associated with improvements in employee wellbeing and performance (84).

Central to employee wellbeing are cultural aspects inherent to workplaces including how organizations are designed and managed. Perceptions of support are a critical factor, while the development of psychologically safe environments are a means to engender such perceptions (85). A psychologically safe environment is one based on mutual trust in which employees feel confident to raise ideas, questions, concerns and make mistakes without fear of punishment, rejection, or humiliation (86) which relates positively to employee wellbeing (87, 88). Although theoretically appealing little is known on the impact of specific interventions in this space.

### Operational and team level: foster growth and wellbeing through a physical and social environment

Social wellbeing focusses on the extent to which individuals have meaningful relationships with others. Social integration, sense of belonging, interdependence, collective consciousness, and collective fate have been identified as key determinants of social wellbeing (89). The kinds of workplace initiatives often employed to target relational enhancements include team cohesion programs (e.g., social events, retreats) and the creation of communal spaces to increase social connection (90, 91). Generally, these programs are delivered as components of broader programs, thus, determining the specific role of singular components is difficult (92). Despite consistent evidence for the importance of co-worker relationships for employee job and life satisfaction (93–95), there is an absence of evidence around *how* to best improve such relationships (96). A recent review found only six studies of interventions in this space and while there is some evidence that interventions increasing the frequency of shared activities could improve worker performance and social environment, findings were inconsistent, and the overall impact on wellbeing remains unclear (90).

Spatial considerations (e.g., natural light, outdoor space) have also been linked with employee wellbeing particularly when employees are involved in the development of such design and placement considerations (38, 97). Open outdoor spaces have been linked with enhanced socialization, active relaxation, and stress reduction (98). Interventions incorporating natural and green spaces have been found to increase positive emotions in the workplace and can lead to long term improvements in functioning and wellbeing (99). Evaluations on the use of indoor plant installations suggest that plants can improve air quality, employees’ perceived comfort, psychological wellbeing, and reduce fatigue (100). Overall, however, physical environmental evaluations tend to be of low quality and meta-analyses are lacking. Furthermore, studies that do exist tend to focus exclusively on office environments.

### Job level: design and re-design jobs to build positive emotional states

There is considerable overlap between strategies to protect and those to promote at a job level, that is, practices which eliminate or successfully mitigate psychological hazards can often also promote positive wellbeing (101, 102). For instance, the SMART work design model aims to enhance workplace wellbeing and thriving within workers beyond simply minimizing hazards to reduce risks (48).

A review of interventions aimed at promoting employee health more broadly through altering working conditions (e.g., work time, intensity, job demands/control) reported significant positive effects in approximately half the studies included (103). Success rates were greater for comprehensive interventions addressing material, organizational, and working time-related conditions simultaneously. A review of healthcare-based interventions to facilitate “sustainable jobs” found that critical components included the enforcement of health and safety obligations, improvements in the workers’ compensation process, the provision of flexible work arrangements, and the integration of employee participation in decision-making (104).

Autonomy at work is strongly related to subjective wellbeing, specifically satisfaction with life and positive affect and is associated with a reduced negative affect (105). Autonomy is closely linked to job control and flexible working conditions, which also contribute to a range of positive health effects (106–108). Similarly, initiatives to improve self-efficacy, mastery (109) and reward/recognition systems (110, 111) are likely to have beneficial effects on employee wellbeing. Few available interventions have been evaluated from a targeted wellbeing perspective, with most instead focused on occupational outcomes (e.g., job performance, turnover intentions).

Job crafting is a means to build autonomy that can circumvent impracticalities of designing jobs to fit all employees (112). Through job crafting, employees can modify tasks and roles to suit their optimal ways of working (113–116). This form of job design is distinctive in that it takes a “bottom-up,” employee-initiated approach, rather than the traditional “top-down” approach in which managers/employers create jobs and roles (117, 118). Job crafting has been linked to increased employee health, job satisfaction, and engagement (119–121). A recent review highlighted the mediating role that job crafting may play in relation to transforming social resources into improved work outcomes (122). However, as this intervention is less prescriptive, there can be unintended consequences stemming from particular decisions employees make in the crafting process (119, 123).

### Individual level: offer programs to improve employee wellbeing

Individual wellbeing interventions usually have a dual function, both increasing positive psychological states and contentment, and reducing negative outcomes such as burnout and absenteeism (124). There is evidence that, in addition, to enhancing mental health directly, such programs can moderate the impact of job stressors (125).

Most individual employee wellbeing interventions utilize physical activity, mindfulness or meditation, positive psychology, or resilience training principles. A recent review found that psychological interventions (e.g., mindfulness and CBT-based approaches) were among the most consistently high-performing interventions to improve worker wellbeing (126). Mindfulness-based programs are among the most widely implemented and evaluated in the workplace. Such programs have been shown to increase positive affect, improve overall wellbeing, life satisfaction, and resilience (126–130). Further findings reported additional benefits to stress and mental health (131). A review of meta-analyses found universally-delivered mindfulness and contemplative interventions yielded a large effect on subjective wellbeing (55). Mindfulness-based interventions also showed a moderate improvement in self-compassion. Compared to mindfulness interventions, CBT-based interventions showed slightly smaller but significant positive effects on wellbeing. Among selectively-delivered programs, psychosocial interventions of this kind had small-to-moderate effects on optimism, self-efficacy, and resilience, and a large effect on positive emotions.

Positive psychology interventions tend to be gratitude- or mindfulness-focused and have also been associated with small-to-moderate effects on work engagement, job performance, and perceived job stress (132). Evidence suggests further positive associations with wellbeing, job and life satisfaction, self-compassion, relaxation, and resilience as well as negative associations with symptoms of depression, anxiety, burnout, and general distress (133). Meyers and colleagues (134) found strong evidence for workplace positive psychology interventions in enhancing employee wellbeing. Resilience interventions based on a combination of CBT and/or mindfulness techniques have also been recommended to both improve wellbeing and reduce the risk of mental ill health (135). Reviews of such resilience trainings indicate positive effects on subjective wellbeing and mental health (136, 137), at least in the short-term (138). Similarly, there is some evidence for the usefulness of CBT solution-focused coaching (and goal setting) (139). There is also emerging evidence exploring the use of spiritual interventions (particularly in healthcare settings) for improving quality of life and wellbeing (140). However, largely these studies are predominantly yoga or mindfulness/meditation based (141).

Several reviews exploring the efficacy of physical health or exercise programs at work focus on productivity outcomes (142–144). Although there is also promising evidence that these interventions (e.g., yoga, walking) can be effective in improving wellbeing, inconsistent findings and low study quality limit conclusions (143, 145). There is also some support for music and art-based interventions on psychological wellbeing among healthcare workers (146). However, evidence tends to be of lower quality.

## Respond

Workplaces have in important role in responding to psychological distress and mental illness of employees. Organizations can support workers who experience mental health problems by building appropriate systems, facilitating care, and offering appropriate adjustments and programs to staff. As with the Protect pillar, in most jurisdictions there are legislated requirements such as workers compensation, discrimination, privacy, and workplace relations laws that direct some aspects within this pillar.

### Systems and policy level: incorporate systems to respond to ill health

How an organization responds to employees showing signs of distress or mental ill-health is in part directed by the broader regulatory framework within which it operates. Relatedly, equity, diversity and inclusion practices are pertinent (e.g., destigmatization). A systematic review of international guidelines found implementation of workplace mental health strategies was often impacted by inconsistent language and a lack of consultation with diverse populations (147). To help comply with legal and ethical requirements, organizations should recognize and address the unique mental health needs related to diversity (e.g., cultural, gender, sexual orientation, ability). While anti-discrimination policies, inclusive hiring practices, and the provision of diversity training is also critical.

Early identification of mental health problems to facilitate care is essential. Wellbeing checks or mental health screening tools are regularly used by organizations to identify those in need of follow-up and intervention. While some early studies indicated that mental health screening in the workplace hold value in improved employee health (148), others have not been able to replicate such findings (149, 150). Further, ineffective screening has the potential to cause harm (151). Most recent findings suggest this kind of screening to only have benefit when linked to enhanced access to treatment (26). As such, currently mental health screening programs have not been recommended in international guidelines for workplaces (2). Although education around care options is important to empower and deliver care, universal approaches followed by advice or provision of referral options is likely to be ineffective in facilitating help-seeking. This is distinct from assessment in the course provision of care or where facilitated access to care occurs which is a necessary component of treatment.

Return-to-work (RTW) planning is generally considered central to recovery and successful reintegration of employees experiencing psychological injury (2). Planning should consider clarity of roles, alignment of worker-employer expectations, advocacy provided by the RTW coordinator, support for the worker’s psychological wellbeing, and the literacy of supervisors and colleagues (152). A recent UK-based review explored evidence for frequently used workplace adjustments related to psychological injury recovery (153). These included adjustments to work schedules, roles and responsibilities, work environment, policy changes, support and assistance, and redeployment. Out of these adjustments, only the work schedule and support and assistance recommendations had evidence of associated health outcomes. Furthermore, despite many workplace adjustments consistently being perceived as effective by staff and stakeholders, adequate testing is lacking and significant barriers to accessing and implementing workplace modifications exist (153).

An Australian rapid review (154) highlighted key themes among programs designed to enable recovery at work or RTW. Themes included the importance of destigmatization, mental health policy, recognition of early warning signs, addressing harms, collaboratively planning (with individual/employer/healthcare provider/case manager), and sustained communication during absence and upon return. While acknowledging a lack of clear evidence for the effectiveness of identified themes, the review recommended that flexible working arrangements, workplace modifications, setting of realistic goals and job expectations, identification of RTW barriers and facilitators, and maintaining trust and confidentiality between the employee and organization should be included in recovery at work or RTW plans.

### Operational and team level: facilitate help-seeking and provide a supportive recovery environment

Operational or team strategies to respond to mental ill health tend to focus on worker- and illness-directed interventions and initiatives to facilitate help-seeking and support recovery. Some of the best evidence for tools to support this process is associated with skill-based training for managers (2). Supervisor support is consistently associated with a range of positive outcomes after a psychological injury at work, including reduced symptoms of poor mental health (155). Evidence indicates that training which focuses on enhancing manager confidence and teaching them new skills in having mental health focused conversations generates improved rates of supportive managerial behaviors (36). There is also evidence from controlled trials that such training also results in reduced rates of work-related sick leave for employees experiencing mental health problems improvements in mental health symptoms at the level of employees have not yet been clearly established (36).

Mental health awareness and anti-stigma programs are also commonly used tools that aim to improve knowledge about mental ill-health and reduce stigmatizing attitudes and discrimination. Evidence suggests that such interventions can have a small positive effect on mental-health knowledge, attitudes, and self-reported supportive behavior, confidence, and readiness-to-help (156–158). There is, however, only limited evidence of sustained improvements of behavior change are mostly derived from subjective self-reports rather than objective observations. Furthermore, how such changes may improve individual symptoms is seldom examined. Relatedly, Mental Health First Aid (MHFA), attempts to teach recognition and response to mental health problems/crises to facilitate help-seeking. This type of training has been associated with improvements in knowledge, attitudes, symptom/sign recognition, and confidence in management of distressed workers (156). MHFA training has also been associated with improvements in reported intentions and provision of support, however, changes in observed behaviors and quality of provided support as well the magnitude of MHFA training impact on any mental health outcomes remain unclear (159).

Peer support programs—in which a small group of specially trained employees provide wellbeing support to other employees—are an emerging form of intervention, especially in high-risk occupations (160, 161). Such programs, which are primarily aimed at improving rates of early help-seeking, have been associated de-stigmatization and fewer perceived barriers to care, with emerging evidence that these shifts may result in reduced sickness absence (162).

### Job level: adjust work to support recovery

RTW policy, supportive environment, active management, RTW plans, and collaborative consultation are considered critical aspects in supporting recovery (163). Labor market integration initiatives including different countries’ laws against discrimination on the basis of disability [e.g., (164)] require employers to provide reasonable adjustments to ensure that employees with disabilities are not discriminated against in the workplace. Job-level adjustments (including, working hour adjustments, staggered return, review of tasks/goals/expectations, private areas, recording of meetings) also apply where recovery takes place in conjunction with work. In addition to adjustments and RTW planning Clayton and colleagues (165) also recommend training and education for staff and managers.

### Individual level: provision of programs to reduce symptoms and illness

Unlike universal or selective approaches, these interventions are aimed at employees who already have symptoms of mental health conditions, with the aim of limiting the progression of illness (“indicated prevention”) and treating those with clinical conditions.

Evidence for indicated prevention for depression supports CBT approaches (55), with some evidence for indicated physical exercise, mindfulness, and resilience training interventions for employees identified as being at-risk of or with early symptoms of a mental illness (166). Digital CBT-based interventions also have been shown to have small effects on depression and anxiety symptoms, while digital stress management interventions have shown promise, but with limited evidence for reducing depressive symptoms and stress (54).

One of the more common interventions offered by organizations to respond to employees experiencing psychological distress at work is workplace counseling, usually facilitated by externally contracted employee assistance programs (EAPs). Workplace counseling can be effective in improving anxiety, stress, and depressive symptoms, but the effectiveness is dependent on the type and quality of psychological intervention provided, which can be very broad and ill-defined (167, 168). More recent reviews on EAPs specifically support the effectiveness of such programs to improve levels of presenteeism and functioning (169). Generally, studies in the area have been of lower quality, with methodological limitations and there is also a lack of meta-analyses to determine overall strength of evidence (170).

## Discussion

This updated Framework seeks to better coordinate strategies and interventions for improving mental health and wellbeing in the workplace and provide recommendations for best practice. There remains a very large gap in knowledge around key aspects of what would constitute effective evidence-based workplace interventions. Some have argued this is in part due to a lack of integration of interdisciplinary expertise and prevention strategies (147), while the complexities of conducting high-quality trials of multifaceted interventions within workplaces are undoubtably also an issue (18). The intricacy of developing tailored interventions for specific workplaces is a further barrier to research practice and scalability. However, recent high-quality studies testing specific workplace interventions suggests that these challenges are not insurmountable and that further research on popular or promising workplace practices must occur.

The evidence base for interventions is clearest at an individual level (as opposed to job, team, or system level) where evaluation studies are least onerous to conduct, involve fewer uncontrollable confounders, investment costs, and demands on organizations. This also highlights a tendency of workplaces to favor changes at the level of an individual worker rather than changes to the work or organization (103). This risk of this approach is twofold. Firstly, it places the onus to remain mentally well on employees and risks overlooking mental health-averse operational or systemic factors that may be present. Secondly, there is a real possibility that the most effective workplace interventions will be at the level of organization-wide or systems-level changes. However, if a robust evidence-base for these is not developed, then best practice cannot be ascertained. It is for these reasons that this new Framework incorporates advice based on both intervention studies and robust, evidence-based theory. Difficulties in conducting trial-based research may require reassessment of the value of traditionally less methodologically rigorous means of evaluation, including high quality observational evidence (e.g., triangulation, natural experiments) (171–173).

In light of an increasing appreciation for the role and benefits of maintaining mentally healthy workplaces (2), these efforts can no longer be considered tokenistic or relegated to individualized, disconnected programs. Although it is important to evaluate specific components to determine the key elements of success, at the level of implementation, holistic multi-level approaches are required that support all workers no matter where they fall on the mental health spectrum, involving all levels of an organization.

It should also be acknowledged that a multitude of external aspects, including broader regulatory and policy frameworks, health and community services, welfare systems, individual lifestyle factors, social networks, and socioeconomic and biological antecedents, influence employee mental health and wellbeing and can impact what workplaces can do to support workers. It is also important to consider both individual disorders and the impacts of comorbidities, both with other mental health conditions, substance use, and physical health conditions and the additional complexities such comorbidities can present. It must therefore be stressed that employers are not solely responsible for employee mental health but are responsible for providing a workplace as free from recognized hazards to mental health and wellbeing as feasible, and for providing a non-discriminatory work environment that promotes wellbeing and recovery from any mental health symptoms that arise. Furthermore, there are important considerations relating to external systems that can inhibit or facilitate action in this space. Compensatory pathways, for instance may provide significant barriers to the recovery process (174). For instance, the definitions around “injury events” and explicit requirements relating to workplace factors and disease causation/contribution differ in different regions, which will impact recovery and return-to-work. The implementation of certain comprehensive programs may also face barriers and challenges during where these external factors are involved (175). In addition, mandatory reporting frameworks and potential career harms of reporting in different roles and industries may impact capacity to provide resources or care options to those in need. It is critical that policy makers and organizations consider the potential stigma and consequences of these factors and appropriately balance worker safety with confidential care options promoting early intervention.

One of the most difficult aspects in providing strong business level recommendations is the heterogenous nature of—not only industry sectors—but individual businesses within a sector. Employers must therefore consider their individual business circumstances, current practices, and risk factors specific to their workplace. The updated Framework looks to categorize specific strategies on different levels of an organization to aid identification of appropriate measures. It should be reiterated that many interventions do not discretely incorporate just one element, address just one pillar or level, or target a single outcome. This segmentation is meant to aid in the identification of appropriate strategies and gaps in an organization’s existing approach. In some cases, particularly at the individual level, there is significant overlap of strategies. Segmentation is useful to highlight where these approaches have differential impact but there tends—unsurprisingly—to be expansive benefit not limited to protect, promote, or respond in isolation. Best evidence presented here, suggests mindfulness and physical health programs may form useful universal protective programs, which also promote wellbeing, while most evidence for CBT programs focuses on symptom reduction (i.e., indicated care). Peer support, similarly, is an example of an intervention which can hold protective, responsive, promotional benefit and future research is needed to explore specific benefits of such programs.

A critical gap in the scientific literature is a lack of information surrounding comparative (cost-)effectiveness, uptake, and acceptability of interventions. This gap is especially pronounced for smaller non-sedentary workforces or workforces in resource-poor settings that do not reflect the generally large white-collar organizations in high-income countries where the evidence base has typically been developed. As the costs of managing workplace mental health issues exceed the costs of prevention measures (176), action in this regard is essential. Generalized OECD advice recommends that employers establish clear policies and conduct organizational psychosocial risk assessments, upskill line managers to increase awareness and competence of mental health-related matters, and support RTW for those on mental health-related sick leave (177). Implementation services can be employed to aid with taking necessary steps, for example, employers could consider liaising with evidence-based consulting services to design and implement initiatives appropriate to circumstances specific to their organization (178). Where organizational resources are not substantial, for example, in smaller businesses, strengthening interpersonal support through relationship building between managers and staff may be critical to aid employee health and wellbeing (28).

## Conclusion

Workplaces have great potential as a facilitator of improving population mental health. While traditionally studies of the links between work and mental health have focused on the role work/workplaces can have in precipitating mental ill health, there is increasing evidence that well-designed and well-managed work can enhance mental health. Ideally, workplaces should be able to promote positive wellbeing, mitigate risk, and to address symptoms of poor mental health at all stages of symptom severity. This goal, however, can only be realized if employers are equipped with the knowledge and access to the resources they need to make positive changes that are appropriate for their specific business and workforce. To this end we have revisited our earlier Framework to Create Mentally Healthy Workplaces (18). The updated Framework provides an overview of different interventions and evidence-based principles to support the wellbeing and mental health of employees. This model clearly defines the types of interventions that are required to create a mentally healthy workplace at the level of systems, operations, job, and individual employee. While the updated Framework presents strategies and initiatives based existing literature or evidence-informed principles of how to protect, respond to, and promote mental health in the workplace, it will need to be applied on a case-by-case basis, by considering what is achievable or relevant for each individual business. A systematic, coordinated, and considered approach to mental health and wellbeing that looks to prevent psychological harm, reduce psychological hazards, support individual recovery, and value employee wellbeing is critical to creating workplaces where employees are healthy and can thrive.

## Author contributions

MD: Conceptualization, Data curation, Formal analysis, Investigation, Methodology, Project administration, Supervision, Visualization, Writing – original draft, Writing – review & editing. SS: Project administration, Conceptualization, Visualization, Writing – original draft, Writing – review & editing. LT: Writing – original draft, Writing – review & editing. NG: Methodology, Supervision, Writing – original draft, Writing – review & editing. AG: Visualization, Writing – original draft, Writing – review & editing. KP: Writing – original draft, Writing – review & editing. VD: Writing – original draft, Writing – review & editing. ES: Investigation, Methodology, Writing – original draft, Writing – review & editing. AL: Supervision, Writing – original draft, Writing – review & editing. SH: Conceptualization, Funding acquisition, Methodology, Supervision, Visualization, Writing – original draft, Writing – review & editing.
